# Lung ultrasound and supine chest X-ray use in modern adult intensive care: mapping 30 years of advancement (1993–2023)

**DOI:** 10.1186/s13089-023-00351-4

**Published:** 2024-02-12

**Authors:** Luigi Vetrugno, Daniele Guerino Biasucci, Cristian Deana, Savino Spadaro, Fiorella Anna Lombardi, Federico Longhini, Luigi Pisani, Enrico Boero, Lorenzo Cereser, Gianmaria Cammarota, Salvatore Maurizio Maggiore

**Affiliations:** 1 Department of Medical, Oral and Biotechnological Sciences, University of Chieti-Pescara, Chieti, Italy; 2 Department of Anesthesiology, Critical Care Medicine and Emergency, SS. Annunziata Hospital, 66100 Chieti, Via Dei Vestini Italy; 3 Department of Clinical Science and Translational Medicine, ‘Tor Vergata’ University of Rome, Rome, Italy; 4 Anesthesia and Intensive Care 1, Department of Anesthesia and Intensive Care, Health Integrated Agency of Friuli Centrale, Piazzale S. M. Della Misericordia 15, 33100 Udine, Italy; 5 Department of Translational Medicine, Anesthesia and Intensive Care Unit, University of Ferrara, Ferrara, Italy; 6 Institute of Clinical Physiology, National Research Council, Lecce, Italy; 7 Anesthesia and Intensive Care, Department of Medical and Surgical Sciences, “Magna Graecia” University, Catanzaro, Italy; 8 Mahidol Oxford Tropical Medicine Research Unit, Bangkok, Thailand; 9 Intensive Care Unit, Miulli Regional Hospital, Acquaviva Delle Fonti, Italy; 10 Anesthesia and Intensive Care Unit, San Giovanni Bosco Hospital, Turin, Italy; 11 Institute of Radiology, Department of Medicine, University of Udine, University Hospital S. Maria Della Misericordia, Azienda Sanitaria-Universitaria Friuli Centrale (ASUFC), Udine, Italy; 12 Anesthesia and Intensive Care, Department of Translational Medicine, Eastern Piedmont University, Novara, Italy; 13 Department of Innovative Technologies in Medicine and Dentistry, Gabriele d’Annunzio University of Chieti-Pescara, Chieti, Italy

**Keywords:** Lung ultrasound, Chest X-ray, Critically ill patient, Intensive care, Ionized radiation, Cost reduction

## Abstract

In critically ill patients with acute respiratory failure, thoracic images are essential for evaluating the nature, extent and progression of the disease, and for clinical management decisions. For this purpose, computed tomography (CT) is the gold standard. However, transporting patients to the radiology suite and exposure to ionized radiation limit its use. Furthermore, a CT scan is a static diagnostic exam for the thorax, not allowing, for example, appreciation of "lung sliding". Its use is also unsuitable when it is necessary to adapt or decide to modify mechanical ventilation parameters at the bedside in real-time. Therefore, chest X-ray and lung ultrasound are today's contenders for shared second place on the podium to acquire a thoracic image, with their specific strengths and limitations. Finally, electrical impedance tomography (EIT) could soon have a role, however, its assessment is outside the scope of this review. Thus, we aim to carry out the following points: (1) analyze the advancement in knowledge of lung ultrasound use and the related main protocols adopted in intensive care units (ICUs) over the latest 30 years, reporting the principal publications along the way, (2) discuss how and when lung ultrasound should be used in a modern ICU and (3) illustrate the possible future development of LUS.

## Introduction

Lung ultrasound (LUS) is an imaging modality that might impact the physician’s decision-making after a patient’s clinical evaluation and accelerates management changes, such as adjustment of ventilatory setting, fluid therapy, patient’s position (supine vs prone), antibiotic management, chest drainage, thus promoting a "functional approach" potentially leading to an improved patient outcome [1]. In this way, ultrasound has become the fifth pillar of medical examination used by intensive care physicians after inspection, palpation, percussion and auscultation [2].

Although the chest is easily scanned with LUS, by just laying the probe along the intercostal spaces, no tool, including LUS, by itself, can improve patient’s outcome. For this reason, the International Evidence-Based Recommendations for Point-of-Care LUS published in 2012 tried to homogenize the terminology and the techniques used and provided a list of recommendations for a clinical approach to the different illnesses [3].

With the COVID-19 pandemic, LUS has become extremely popular and this was reflected by many publications and by countless theoretical and practical courses, both delivered online and in presence [4]. This narrative review aims to explore the following points: (1) to analyze the advancement in knowledge of LUS signs and the related main protocols used in ICUs over the latest 30 years, reporting the principal publications along the way, (2) to discuss how and when LUS should be used in a modern ICU and (3) to illustrate the possible future development of LUS.

## Evolution of LUS in critically Ill patients

From 1995 the American College of Radiologists recommended daily supine chest X-rays in mechanically ventilated patients with acute cardiac and respiratory problems independently from the underlying pathology [5].

At that time, detecting tubes and central line malposition or the discovery of pneumothorax (PNX) with chest X-ray was responsible for a change in patient diagnosis or management in more than 65% of the cases [5]. However, the new millennium brought a breath of fresh air in critical care medicine and heralded the publication in 2000 of the paper "The Acute Distress Syndrome Network" about lung-protective ventilation strategy [6]. This article produced evidence for the protective effect of low tidal volume (6–8 ml/Kg) and its application worldwide, and this saw a reduction in the incidence of volotrauma.

**Fig. 1:**
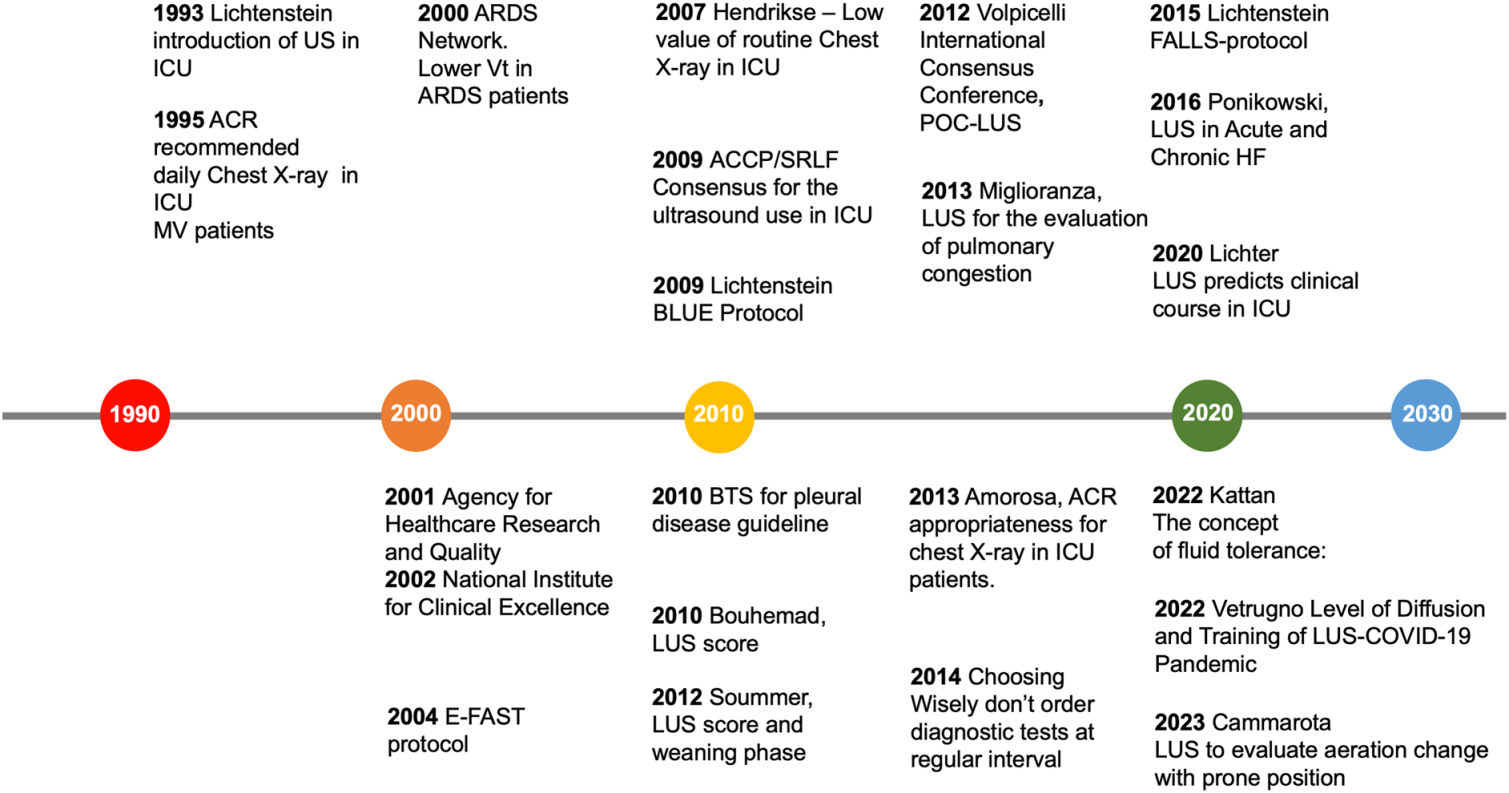
Lung ultrasound vs Chest X-ray Road map. American College of Radiology (ACR), acute respiratory distress syndrome (ARDS), American College of Chest Physicians (ACCP), intensive care unit (ICU), La Société de Réanimation de Langue Française (SRLF), Bedside Lung Ultrasound in Emergency (BLUE) protocol, point of care lung ultrasound POC-LUS, Extended Focused Assessment with Sonography for Trauma (E-FAST), British Thoracic Society (BTS)

Meanwhile, further important events happened in 2001 and 2002: the use of ultrasound for central venous catheter (CVC) placement was promoted in the USA by the Agency for Healthcare Research and Quality as one of the 11 practices to improve patient care [7], and by the National Institute for Clinical Excellence (NICE) in Europe, and this started to reduce the iatrogenic PNX related to blind CVC placement [8]. Many discoveries by Daniel Lichtenstein, the father of modern LUS use in the ICU, saw the dawn in the new millennium between 1995 and 2009, with the description of "lung sliding", a bedside ultrasound sign ruling out pneumothorax (1995), [9] the comet-tail artifact as a sign of “alveolar-interstitial syndrome” (1997) [10], the “lung point”, an ultrasound sign specific to pneumothorax (2000) [11], the “lung pulse”, an early sign of complete atelectasis (2003) [12], and the dynamic air bronchogram, a lung ultrasound sign of alveolar consolidation ruling out atelectasis (2009) [13]. In his pioneering work first published in 1993, Lichtenstein used ultrasound to examine the abdomen, the pleural space, and the femoral vein at the bedside in ICU [14]. The study results were surprising, with ultrasound showing an immediate impact on management in 33 out of 150 patients (22%), influencing the diagnostic workup and directly impacting the therapeutic decision-making approach; and providing promising results to open the way for ultrasound use in the ICU [14]. Figure 1 (left part).

## LUS and consolidation, interstitial syndrome, pneumothorax and pleural effusion

Lichtenstein was also the first to compare lung ultrasound sensitivity, specificity and diagnostic accuracy with auscultation and chest X-ray in patients with lung consolidation [15]. His second most impactful article focused on the "Bedside Lung Ultrasound in Emergency” (BLUE) protocol about the relevance of LUS in diagnosing the etiology of acute respiratory failure [16]. With the previously described signs of lung pulse, an early sign of complete atelectasis (2003), and the dynamic air bronchogram (2009), it is easy today to recognize pneumonia as a cause of acute respiratory failure [12]. In this algorithm, for example, comet-tail artifacts—B-lines today—helped differentiate cardiogenic pulmonary oedema from exacerbation of chronic obstructive pulmonary disease (COPD) with a sensitivity of 100% and a specificity of 92% [16]. After this discovery, in 2013 The European Association of Cardiovascular Imaging Recommendations stated that the absence of multiple bilateral B-lines excludes cardiogenic pulmonary oedema with a negative predictive value close to 100% [17].

On the contrary, B-lines were significantly correlated with a new onset acute congestive heart failure if their number was ≥ 15 per scan. This cut-off could be considered for a quick and reliable assessment of decompensation in outpatients with heart failure (HF) [18]. That was followed in 2016 by the European Society of Cardiology Guidelines stating that for the diagnosis and treatment of acute and chronic heart failure, chest X-ray is only of limited use in the diagnostic work-up of patients with suspected HF and probably most helpful in identifying an alternative pulmonary explanation for patient's symptoms and signs [19].

The diagnosis of PNX with LUS deserves a further separate examination. Considering that supine antero-posterior (A-P) chest X-ray may misdiagnose up to 30% of cases of PNX detected with a computed tomographic (CT) scan, LUS can be extremely useful in everyday clinical practice [20]. In the context of trauma, the case of pneumothorax not detected by plain radiography but later confirmed by CT, was first described in 2001 by Kirkpatrick [21]. In a comparative study, the same author first described the superiority of LUS vs chest X-ray for PNX detection in trauma patients when extending the abdominal ultrasound examination to the lung, applying the Extended Focused Assessment with Sonography for Trauma (E-FAST) protocol in 2004 [22]. In this study, LUS showed double sensitivity compared with chest-X ray (48.8% vs 20.9%), while specificity was high for both diagnostic images 99.6% vs 98.7% respectively. Soldati et al. in 2008, reported that out of 218 trauma patients, 25 showed PNX on CT scans. Taking the diagnostic accuracy of CT scan as the gold standard, the authors compared it with chest X-ray and LUS, and found that only 52% of PNX were revealed by chest X-ray with a sensitivity of 52% and specificity of 100%. In comparison, LUS detected 23 of 25 PNX with a sensitivity of 92% and specificity of 99.4% [23]. These findings have been confirmed today by evidence arising from 3 meta-analyses [24–26].

**Fig. 2:**
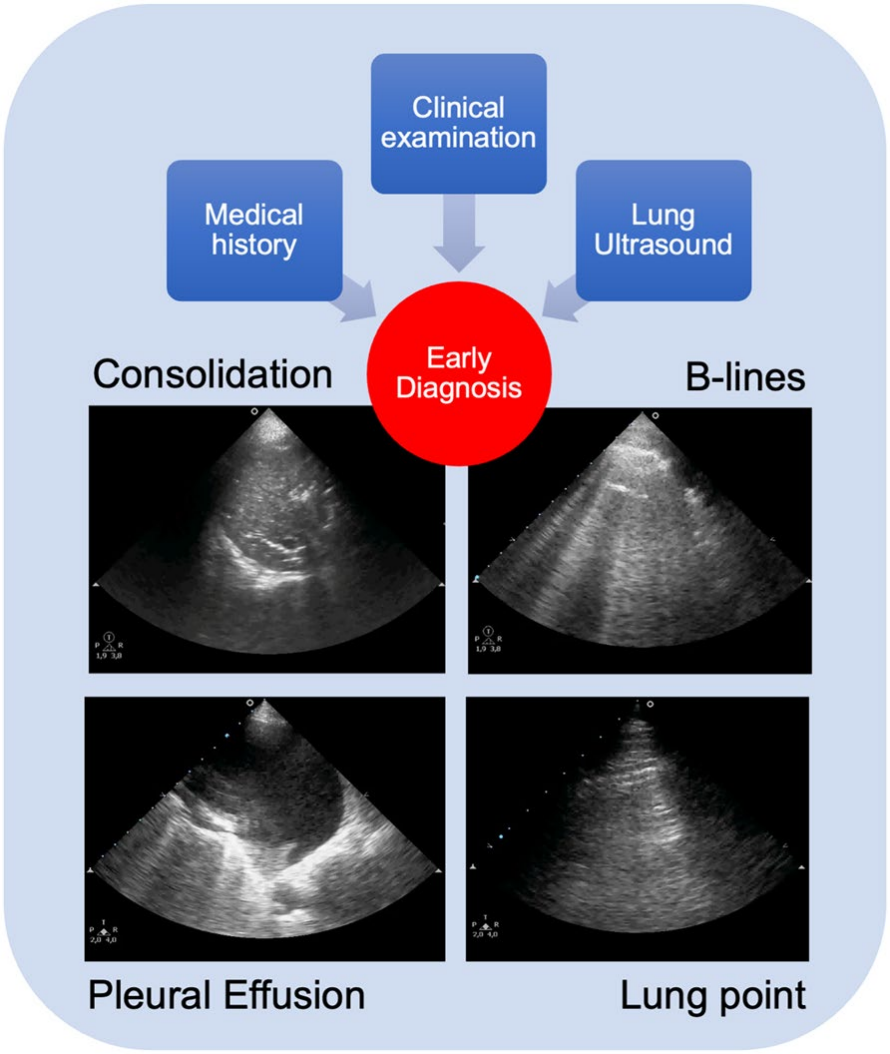
LUS is a valid bedside tool that helps clinicians to reach the right diagnosis also taking into consideration medical history and clinical examination

Pleural effusion (PE) is another example of the superiority of LUS compared to supine chest X-ray in ICU, where patients' physical examination with percussion and auscultation have shown low sensitivity and specificity compared to CT scan as the gold standard [27–30] (Figure 2). In practice, comparing LUS with a supine chest X-ray for pleural effusion produced important findings. In fact, this last becomes abnormal when PE is ≥ 200 mL, obliterating the hemi-diaphragmatic sinus [15, 31]. Furthermore, the possibility of coexisting parenchymal lung opacities further decreases chest X-ray sensitivity, while 'normal' appearances do not exclude the presence of an effusion [15, 29–31]. In 2008, Rocco et al. published a trial comparing bedside chest X-ray and LUS to diagnose PE in trauma patients, finding that LUS was more accurate than chest X-rays [32]. Another study by Xirouchaki et al. comparing the diagnostic accuracy of LUS and bedside chest X-ray in ICU patients showed excellent sensitivity, specificity, and an accuracy of 100%. In contrast, chest X-rays showed suboptimal corresponding values of 65%, 81%, and 69%, respectively [33]. Therefore, evidence suggests that LUS is more accurate for detecting pleural effusion than a supine chest X-ray. However, with ultrasound is not possible to distinguish PE type, i.e., exudate vs transudate, and after 50 years, the report by Light et al. is still the landmark in determining the different origins of effusion [34].

## Estimating pleural effusion and improving accuracy in chest drainage positioning

An essential task of LUS is the possibility of quantifying PE, and for this purpose many formulas exist [35–39]. All the authors in Table 1 found a good correlation between PE volume estimated by their formulas and effective volume measured after drainage. No superiority of one formula over another has been demonstrated to date. The Balik formula has gained popularity for its simplicity by measuring PE volume by multiplying the maximal interpleural distance (D) at lung base with a constant factor (V(ml) = 20 × D (mm)) [37]. The average error of this formula can be calculated at around 158–160 mL. Moreover, the formula overestimates the volume in some conditions, like in tall males with large thoracic circumferences, small effusions under 200 mL, and more significant effusions above 1000 mL [38, 39]. In the context of PE, another important event occurred in 2010 with the publication of the British Thoracic Society (BTS) Pleural Disease Guideline [28], now revisited, in response to the rapid system report of 12 deaths related to chest drain insertion, and 15 cases of serious harmful events between January 2005 and March 2008 [40]. The BTS strongly recommended that all chest drains for pleural effusion should be inserted under ultrasound guidance with small-bore chest drains for all fluid types in the thorax [40, 41]. In this context, the main purpose of the ultrasound is to identify a safe site for fluid aspiration followed by an accurate positioning of the chest drain insertion. A detailed illustration has been previously described [29]. In a recent ICU study, pleural drainage in terms of diagnostic and therapeutic impact has been showed to improve the pre-drainage diagnosis in 91 out of 119 (76.5%) patients, with 62 (52.1%) of these resulting in a complete change in the diagnosis, and 80 out of 137 procedures (58.4%) resulting in a change in treatment. However, extubation success and weaning from non-invasive ventilation (NIV) were not affected by drainage—17.5% vs 30.9% (*p* = 0.13) and 40% vs 66.7% (*p* = 0.38), respectively, as well as no difference was found regarding in-hospital mortality between those treated and not, 27.3% vs 27.3% (*p* > 0.99), respectively [42]. It should also be noted that thoracic ultrasound is only of limited utility in guiding the insertion of a chest drain in the presence of PNX because of the difficulty in obtaining valuable images due to the poor transmission of sound waves through the air. Table 2 [3]

**Table 1:** How pleural effusion with ultrasound is measured, according to different formulas

Authors	How measurement is made (end-expiration)
Vignon et al. [35]	Measured the maximal perpendicular interpleural distance (the distance between the lung and posterior chest wall) at the apex and the lung base and compared the maximal distance with the drained volume
Roch et al. [36]	Mean of 3 distances measured between lung and diaphragm, lung and posterior chest wall at the base, lung and posterior chest wall at the fifth intercostal space
Balik et al. [37]	Measured the maximal interpleural distance (D) at lung base and used the formula Volume (ml) = 16 × D (mm)
Usta et al. in patients after cardiac surgery [38]	Measured the maximal distance between the mid-height of the diaphragm and the visceral pleura in the sitting position while spontaneously breathing
Remérand et al. [39]	Identified the lower and upper intercostal spaces where pleural effusion is visible in supine patients; the distance between these two points was drawn on the patient's skin to establish pleural effusion paravertebral length. The pleural effusion cross-sectional area is manually delineated at the half way point with LUS. The volume of pleural effusion is obtained by multiplying LUS by the cross-section area

Lung ultrasound (LUS

**Table 2:** Comparison of thoracic image characteristics for diagnosis, procedures and monitoring

	Image modalities for documenting the disease	Image modalities after procedures	Image for daily monitoring
(supine) Chest X-ray	Less accuracy compared with LUS and CT scan (–-); Superior to LUS in case of subcutaneous emphysema (+ + +);	More precise for CVC tip location (+ + +); PAC, nasogastric and chest tube positioning (+ + +);	Excessive radiation exposure, risk of patient trauma and PTSD (–-);
LUS	More accurate for consolidation, pleural effusion, interstitial syndrome, and pneumothorax (+ +); Not applicable in case of subcutaneous emphysema (–-);	More accuracy to guide pleural drainage position (+ + +);	Optimal for daily evaluation of the LUS score, radiation free, low cost (+ + +);
CT scan	Gold standard for diagnosis of consolidation, pleural effusion, interstitial syndrome, and pneumothorax and the only one for PE (+ + +);	Gold standard (+ + +);	Not applicable (–-);

*LUS* Lung ultrasound, *CT* Computed tomography, *CVC* Central venous catheter, *PAC* pulmonary arterial catheter, *PTSD* post-traumatic stress disorders

## LUS and fluid tolerance

Detecting fluid responsiveness is an important task in the management of critically ill patients. Moreover, detecting fluid tolerance, defined as the extent to which a patient can tolerate fluid administration without falling into organ dysfunction, is also of paramount importance [43]. Therefore, fluid resuscitation in ICU is a double-edged sword; under-resuscitation with hypoperfusion and overhydration with venous congestion are both dangerous, and they require a careful assessment of what intravenous volume status and fluid tolerance are [44, 45]. We can study fluid tolerance and intolerance with LUS through evaluating the presence of B-lines [46, 47]. In this review, we will emphasize that LUS can better detect interstitial syndrome compared with chest X-ray with an accuracy of 93% [15–46]. As described by Lichtenstein, it is important to know that assessing interstitial syndrome requires the study of the anterolateral zones of the chest as the posterior zone suffers from fluid gravity [48, 49].

**Fig. 3:**
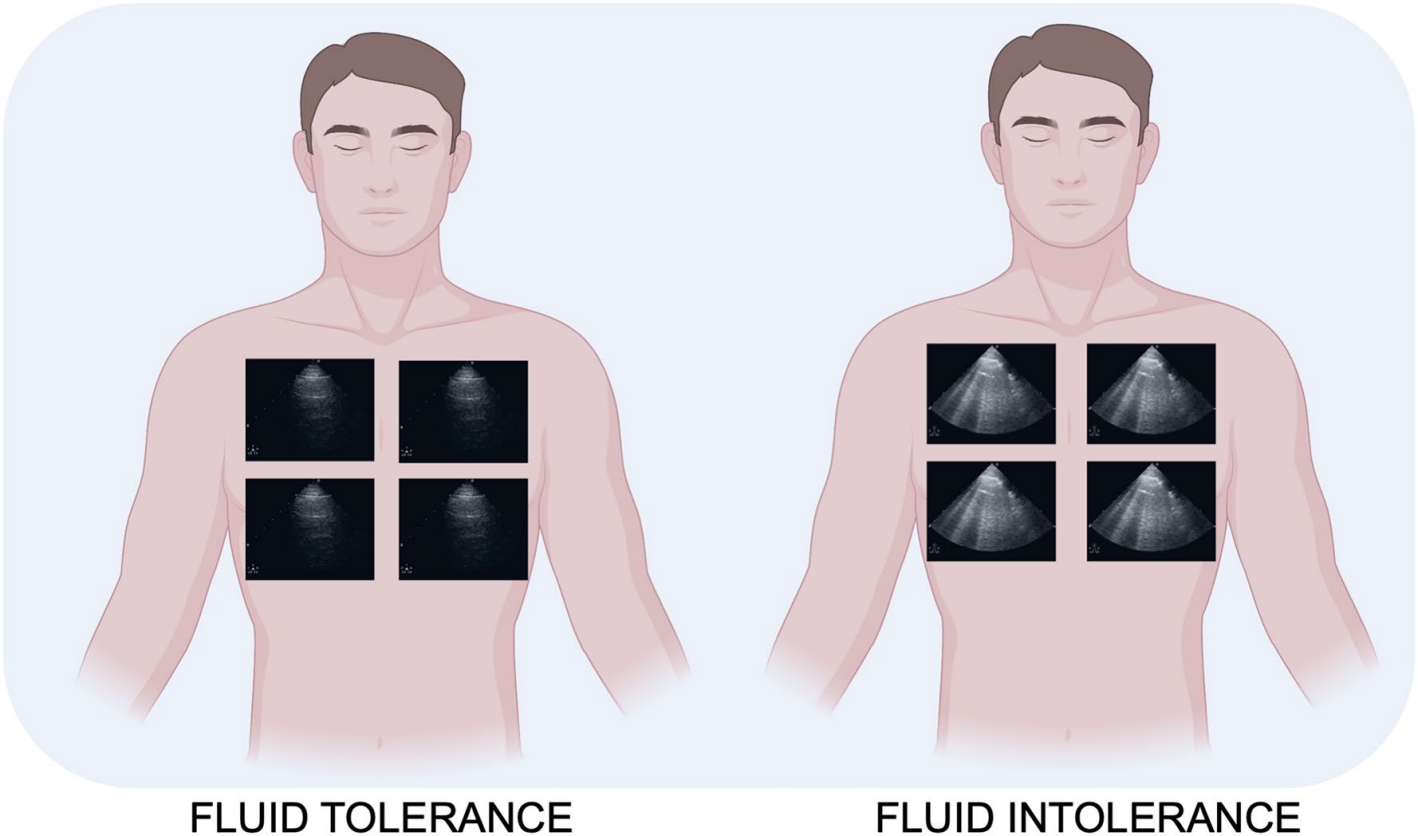
Assessing interstitial syndrome with LUS for fluid tolerance and intolerance requires antero-lateral chest zone exploration as the posterior zone suffers from fluid gravity

Interstitial syndrome suggests cardiogenic pulmonary oedema with a sensitivity of 97% and specificity of 95% when B-profile arises from the base to the apex and the pleural is thin (Fig. 3). Fluids in this case are not recommended [43–49].

Interstitial syndrome is also present with B-lines with anterolateral B-profile in case of isolated or diffuse ARDS. Copetti et al. helped in recognizing interstitial syndrome due to ARDS from pulmonary oedema by describing those pleural lines as thickened with reduced "gliding" in the context of "spared A-lines areas" in cases of ARDS [49]. The optimal fluid management in ARDS patients remains challenging and controversial because it should provide adequate oxygen delivery to the body while avoiding an inadvertent increase in lung oedema. Monitoring B-lines in this setting also requires echocardiographic evaluation and, if appropriate, an advanced invasive hemodynamic monitoring tool [50],because these patients can also develop a low cardiac output (CO) state with a reduced left ventricular ejection fraction (EF%) or isolated diastolic dysfunction (DD), respectively, in 39% and 20% [51, 52]. Volpicelli et al. first noted that B-lines correlate with extravascular lung water (EVLW) measured with the invasive monitoring PiCCO tool [53]. In that way, restrictive strategies, including fluid restriction guided by the monitoring of extravascular lung water, have been shown to improve oxygenation and reduce mechanical ventilation duration significantly, but with no significant effect on mortality [54, 55]. On the contrary, the absence of B-profile—“lung tolerance”—in patients with shock is a clear indication for intravenous fluids [43, 56, 57]. A new concept about organ congestion known as the venous excess with ultrasound score (VExUS) evaluating lung, liver and kidney is also developing. VexUS is outside the scope of this review and requires further literature evidence [47].

## Lung ultrasound score in ICU

Lung ultrasound can scan the lung surface through the anterior, lateral and posterior areas giving a score based on the different aeration patterns from 0 to 3 from the best to the worst as follows: A-lines plus sliding = 0, well-separated B-lines = 1, coalescent B-lines = 2, and for C-pattern, consolidation = 3 [58]. An increase in score indicates a decrease in aeration and vice-versa. LUS score is feasible and easily obtained at the bedside to understand the effect of modification of the ventilation parameters, of patient’s positioning (supine vs prone), and of weaning outcome [59, 60]. This score was first proposed by Soummer et al. in a work that highlighted its use during the weaning phase from mechanical ventilation. LUS changes during a spontaneous breathing trial accurately predict post-extubation distress and the first time a switch in LUS use from diagnostic to monitoring was proposed in 2012 [61]. In 2010 Via et al. showed the LUS score to be reliable in evaluating lung aeration changes in patients who underwent whole lung lavage [62]. Bouhemad et al., in 2010, studied antibiotic-induced pulmonary reaeration in ventilator-associated pneumonia. The authors compared CT scan, LUS score and chest X-ray regarding reaeration of lungs following 7 days of antimicrobial therapy [63]. The authors use a 12 regions exam between days 0 and 7. They found that an ultrasound score > 5 was associated with a computed tomography reaeration > 400 mL and successful antimicrobial therapy. While an ultrasound score < 10 was associated with a loss of computed tomography aeration > 400 mL and a failure of antibiotics. Computed tomography and ultrasound lung reaeration showed a highly significant correlation (Rho = 0.85, *p* < 0.0001), while chest X-ray was inaccurate in predicting lung reaeration changes [63]. With the SARS-CoV2 pandemic leading to interstitial pneumonia characterized by superficial and subpleural lung lesions, interest in the LUS score has rapidly spread. The most famous study in this area was conducted in Israel in a medical ward and intensive care setting. In 280 patients, Lichter et al. found that LUS score predicted clinical deterioration and death [64]. In another study from a Brazilian group, de Alencar et al. found in 180 patients a correlation between the LUS score at admission and the duration of mechanical ventilation, intubation, and the probability of death. This study considered a broader population spectrum admitted to the emergency department with only 74 ICU patients [65]. We also found that COVID-19 patients with a lower LUS score after ICU admission had a better survival rate than those with a high score [66]. Furthermore, we identify four sub-phenotypes: (a) those with clinical improvement independently from the LUS score; (b) those who presented a moderate improvement in LUS score; (c) those who responded very clearly, after mechanical ventilation with a significant reduction in LUS score; (d) those who, while improving their clinical condition, did not show an evident improvement from an ultrasound point of view and presented an apparent worsening in LUS. This could mean that different components can influence the LUS score. For example, underlying cardiac and pulmonary diseases such as HF, COPD, chronic asthma, and the development of new pulmonary fibrosis could be crucial. The fact that some patients showed an immediate improvement in the LUS score after mechanical ventilation probably underlines a misdiagnosed cardiac involvement; on the contrary, patients who showed no improvement in the LUS score could have pulmonary fibrosis [67]. Interestingly, a recent multicenter prospective observational study proposed a new score in non-COVID-19 patients called the LUS-ARDS score [68]. It is based on LUS aeration scores of the left and right lung plus the anterolateral pleural line abnormalities—that score has been compared with the performance of chest X-rays in ARDS patients. The LUS-ARDS score showed an area under the receiver operating characteristics (ROC) curve of 0.90 (CI 0.85–0.95), a comparable performance to the current practice with experienced chest X-ray readers, but with more objectifiable diagnostic accuracy at each cut-off [68].

## Lung ultrasound: basic and advanced skills

The use of ultrasound in ICU was first classified in 2009 by the American College of Chest Physicians (ACCP) and “La Société de Réanimation de Langue Française” (SRLF) [69]. Clinical ultrasound competencies were validated using the Delphi methodology and divided into two main branches: the general critical care ultrasound (GCCU) focusing on the thorax, abdominal and vascular level assessment, and the critical care echocardiography (CCE) with two levels of expertise, basic and advanced. Pleural and LUS were components of the GCCU. For pleural ultrasound, critical steps guided thoracentesis and pleural device insertion, estimating the remaining pleural fluid and identifying intrapleural device placement. LUS was explicitly oriented to discover PNX after the procedure [69]. In 2012, the international consensus conference also introduced the important concept of monitoring lung aeration and de-aeration with LUS scores to quantify the effect of ventilatory strategies [3]. However at the moment, it is difficult to separate LUS knowledge into basic and advanced on a continuum, and citing the recent work of Kraaijenbrink et al., “the high negative predictive value of ruling-out a PNX with lung sliding suggests this is straightforward, but ruling-in a PNX is much more complicated needing a different number of exams to become proficient showing that artifacts have a different learning curve.” [70]. Therefore, the low incidence of pneumothorax makes the recognition of the lung point difficult, with the need for a long training. The same is true for consolidation, an essential skill to differentiate between pneumonia, atelectasis, contusion and pulmonary embolism. It requires advanced skills supporting the idea that competence cannot easily be divided into basic and advanced skills [71, 72].

## Chest X-ray in ICU after 2012–2014

In 2006, Graat et al. first demonstrated in 2,457 daily routine chest X-rays performed in 754 consecutive ICU patients that this imaging modality did not reveal any new predefined significant findings [73]. This work was followed by a trial in which one patient group underwent a supine chest X-ray daily and a second group on demand. The results showed that on-demand chest X-rays did not delay the diagnosis, length of stay, ventilator-free days or increased mortality between the two groups. Subsequently, Oba and Zaza performed a meta-analysis involving 7,078 patients and found that the elimination of daily routine chest X-rays did not affect ICU LOS (WMD = 0.19 days; 95% CI –0.13, 0.51; P = 0.25), hospital LOS (WMD = –0.29 days; 95% CI –0.71, 0.13; *P* = 0.18), ventilator days (WMD = 0.33 days; 95% CI –0.12, 0.78; P = 0.15), ICU or hospital mortality (OR, 1.02;[95% CI 0.89, 1.17; *P* = 0.78 and OR, 0.92; 95% CI 0.76, 1.11; *P* = 0.4, respectively) [74].

Hendrikse et al. analyzed the data on 1,780 daily chest X-rays in 559 hospital admissions, underlining the diagnostic efficacy of daily routine chest X-rays at 4.4% [75]. Following this evidence, the American College of Radiology (ACR) amended its recommendations in December 2011 by assigning a “usually not appropriate” rating with some exceptions to routine daily chest X-rays [76].

In 2014, the category of patients receiving mechanical ventilation was removed from ACR recommendations, and routine chest X-rays in all stable patients in the ICU were categorized as “usually not appropriate.” [77]. And then, “Do not order diagnostic tests at regular intervals (such as every day)”, including daily chest X-rays, was among the top 5 Choosing Wisely list [78]. However, scepticism persists [4, 61]. Figure 1. (Right part).

## Chest X-ray vs LUS cost

Hejblum et al. assessed chest radiographs in mechanically ventilated patients in 21 ICUs; 11 were using daily chest radiographs and 10 a clinical-driven strategy. Four hundred and twenty-four patients received 4,607 routine chest radiographs, and 425 received 3,148 clinical-driven chest X-rays. There was a 32% reduction in the second group with a 35% reduction in chest X-rays and $9,900 per bed per year without any change in the quality of care or safety [79]. Scott et al. showed that by restricting daily chest X-rays in ICU, the average monthly cost decreased from $11,633 before the intervention to $7,348 after the intervention, with a 37% cost saving [80]. Peris et al., after the introduction of LUS to their ICU, showed a significantly decreased use of diagnostic chest X-rays and CT scans by 26% and 47%, respectively, with a 39% cost saving in radiological examination (around €27,000) [81]. The authors also found a trend in the reduction of CT scans. We also analyzed the cost of chest X-rays after implementing LUS use showing a reduced related cost of 57% (€22,104) without affecting patient outcomes. Significantly, the number of CT scans remains the same [82].

## Limitations of chest X-ray vs LUS

The limitations of bedside portable chest X-ray should also be highlighted in terms of image quality and, more importantly, the inability to accurately diagnose critical causes of dyspnoea, such as pleural effusion, pneumothorax, pulmonary edema and embolism. With a chest X-ray, 10% to 25% of pleural effusion can be misdiagnosed entirely, and 30% of pneumothoraxes are not visible because air moves up and medially between the lung and the heart, and only after filling these spaces free air gather the usual apical-lateral position. Chest X-ray is moderately specific (specificity 76%, 83%) but not sensitive enough (67–68%) for diagnosing heart failure, where the crucial exam is echocardiography. Chest X-ray also showed low sensitivity for pulmonary embolism [83]. Although LUS, as shown above, has several advantages, it has relatively lower sensitivity compared to chest CT. Only 70% of the lung surface can be explored, which explains the relatively low sensitivity to detect intra-parenchymal pneumonia not adherent to the pleural surface. LUS may be more challenging in obese patients due to the thickness of their ribcage and soft tissues. However, the primary enemy of LUS is that subcutaneous emphysema prevents the propagation of the ultrasound beams to the subpleural lung parenchyma [84].

## Chest X-ray and irradiation

When dealing with radiological imaging techniques, it is imperative not to separate appropriateness from radioprotection issues. Specifically, critical care physicians and radiologists must always be mindful of the risk of exposure-induced biological effects resulting from ionizing radiations. Such effects are considered stochastic in diagnostic imaging, i.e., they can randomly derive from damage in a single cell, possibly resulting in cancer in the exposed individuals and hereditary diseases in their descendants [85]. For such effects, the International Commission on Radiological Protection (ICRP) has adopted a linear-no-threshold dose–response relationship, meaning that increasing the dose corresponds to an increased event frequency but not severity [86]. In other words, the excess risk of stochastic adverse effects is directly proportional to the radiation dose received, without any threshold below which there is zero excess risk [86]. While data on the rate of radiation-induced cancer are more solid in cases of high-dose exposures, such as those observed in cohorts of atomic bomb survivors, [86] there is a lack of clear evidence regarding the stochastic cancer risk for low-dose exposures, i.e., those below an effective dose of 100 milliSievert (mSv). As a point of reference, a single posteroanterior X-ray is estimated to deliver a dose of 0.02 mSv, whereas a chest CT is equivalent to 300–400 CXRs (about 6–8 mSv) [87]. While the burden of stochastic risk associated with radiological diagnostic imaging may be overestimated, it is prudent to err on the side of caution when addressing radiation exposure issues [88]. Therefore, radiation exposure should always be kept to a minimum. Indeed, even when the risk inherent to a given exposure is shallow, using close reiterated radiological examinations in patients with chronic conditions may lead to non-negligible cumulative radiation exposure. In addition, the patient’s biological risk for a given dose is highly dependent on age and gender, with children and women at greater risk, thus making the ICRP-promoted “as low as reasonably achievable—ALARA” recommendation even more important [89]. This cautious theoretical approach translates into the assumption that one can define chest X-ray and CT scan use in ICU diagnostic practice as appropriate only when coupled with increasing knowledge of patient radiation-inherent risks and benefits. Indeed, the responsible use of radiological examinations requires an awareness that, while they can be life-saving, alternative imaging techniques that do not involve ionizing radiation, such as LUS, should be preferred when the desired information can be obtained with comparable accuracy [90].

## Further direction with LUS in ICU

As stated before, LUS use in ICU is now relevant to providing image modality that, integrated with patient clinical information, may impact physician decision-making and accelerate management changes in terms of adjustments of ventilatory setting, fluid therapy, patient’s position (supine vs prone), antibiotic management, and chest drainage [91] It is not possible to neglect LUS use today in ICU when also considering the economic and environmental impact over chest X-ray [92]. Many studies will be soon available showing the usefulness of this instrument in patients with pneumonia, ARDS or to evaluate patient weaning from mechanical ventilation. LUS is an operator-dependent exam, and the quality of images may vary depending on the technique and skill, which requires a steep learning curve, making challenging ultrasound studies replications and generalizable conclusions on its utility. Developing dedicated algorithms with artificial intelligence (AI) that automatically evaluate LUS video acquisition through real-time feedback could help in this direction [93]. At the same time, another exciting area of research could be using contrast-enhanced ultrasound (CEUS) imaging to characterize consolidations, helping choose optimal patient treatment and reduce the need for radiation exposure regarding CT scan exams [94]. Finally, in the ICU, experimental studies investigating LUS sliding velocity as a sign of lung dissection could improve ventilation setting and PEEP titration during mechanical ventilation [95].

## Conclusion

In this review, we have analyzed the progress of LUS and the decline of chest X-ray use in ICU over the latest 30 years, highlighting the turning points brought about by new discoveries. LUS has shown to be an essential tool in enhancing patients’ safety and helping clinicians reach a bedside diagnosis and monitor patients over time. We have also discussed the problem related to the economic point of view and patients’ radiation exposure so that a supine chest X-ray could be reduced to the minimum while LUS should be incorporated into the ICU physician’s armamentarium. We should consider that at the present time, the major limitation is the need for a reasonable number of expert supervisors in the ICU team, together with the homogenisation of the image acquisition modality and the standardization of the exam’s report. In the near future, with the widespread use of technologies, it could be possible to review and discuss the LUS images at the patient’s bedside and use these images to monitor progress throughout the patient’s journey in the ICU.

## Data Availability

Not applicable.
